# Dryer Vents: An Overlooked Source of Pollution?

**DOI:** 10.1289/ehp.119-a474a

**Published:** 2011-11-01

**Authors:** Rebecca Kessler

**Affiliations:** Rebecca Kessler, based in Providence, RI, writes about science and the environment for various publications. She is a member of the National Association of Science Writers and the Society of Environmental Journalists.

It’s easy to tell when your neighbors are doing the wash: just sniff the outdoor air for the scent of laundry products. In a new study, researchers have identified components of those scented emissions that are classified as hazardous air pollutants and known or probable carcinogens.^1^ The bottom line, says first author Anne Steinemann, is that “there are hazardous chemicals coming out of dryer vents.” However, very little research has examined whether exposure to these chemicals in the context of dryer-vent emissions has adverse health effects.

Toxicological studies have shown that many individual fragrance ingredients are safe at the concentrations used in consumer products.^2^ However, exposure to scented products has been documented to cause irritation of the eyes and airways, contact dermatitis, migraines, and asthmatic reactions, particularly in sensitive individuals.^3^^,^^4^^,^^5^^,^^6^^,^^7^ According to one 2000 article, mice exposed to emissions from five fabric-softener products experienced sensory and pulmonary irritation and airflow limitation.^8^ And in a 2005–2006 national telephone survey, Steinemann and colleague Stanley M. Caress found that 10.9% of 1,058 U.S. respondents reported being irritated by the scent from laundry products vented outdoors.^9^

In the current study, Steinemann, a professor of civil and environmental engineering and public affairs at the University of Washington, and colleagues identified compounds in a top-selling brand each of laundry detergent and dryer sheet by conducting gas chromatography/mass spectrometry (GC/MS) headspace analysis—sampling the air above 2 g of product placed inside a sealed glass container for 24 hours. Using the washing machine and dryer at two Seattle-area homes, the investigators ran a total of six loads of new, pre-rinsed organic cotton towels. They conducted GC/MC analysis on dryer-vent emissions from loads that used no laundry products, detergent only, and detergent followed by two dryer sheets.

The investigators identified a total of 29 unique volatile organic compounds (VOCs) in the dryer-vent emissions. Ten VOCs identified in the headspace analyses also appeared in the emissions when laundry products were used. The U.S. Environmental Protection Agency (EPA) classifies seven of the VOCs found in dryer-vent emissions—acetaldehyde, benzene, ethylbenzene, methanol, *m/p*-xylene, *o*-xylene, and toluene—as hazardous air pollutants.^10^ The EPA considers acetaldehyde a probable human carcinogen^11^ and benzene (found in two dryer-vent emission samples) a known human carcinogen.^12^

The three VOCs present at the highest concentrations were acetaldehyde at a maximum of 47 µg/m^3^ (0.03 ppm), acetone at a maximum of 36 µg/m^3^ (0.02 ppm), and ethanol at a maximum of 50 µg/m^3^ (0.03 ppm). These values exceeded average annual ambient concentrations for the local area by more than 10 times for acetone and more than 25 times for acetaldehyde.^1^ Although an actual exposure assessment would need to be conducted before any predictions could be made about health effects of dryer-vent emissions, the values are well below Acute Exposure Guideline Levels established by the EPA for these chemicals (200 ppm/10 min for acetone and 45 ppm/10 min for acetaldehyde).^13^ For ethanol the American Industrial Hygiene Association set a comparable guideline of 1,800 ppm/1 hr.^14^

Charles Weschler, a chemist specializing in air pollutants with the University of Medicine and Dentistry of New Jersey who was not involved with the current study, called it “a nice demonstration that indoor activities can potentially make a contribution to outdoor pollutants.” It also makes a persuasive case that some of the VOCs in the vent emissions probably did come from the laundry products, he says. “You see them in the headspace analysis, and you turn around and see some of these same constituents in the emissions.”

Weschler says the reported acetaldehyde concentrations are high enough to cause concern about sensory irritation if dryers are vented indoors, as sometimes happens intentionally with the use of indoor dryer-vent kits, or unintentionally if equipment malfunctions.

Ladd Smith, president of the industry group the Research Institute for Fragrance Materials in Woodcliff Lake, New Jersey, which issued a press release criticizing the study,^15^ is much more skeptical. He expresses concern that the study points a suggestive finger at the laundry products without proving that’s where the VOCs came from or ruling out ambient air, the towels, or the dryers themselves as potential sources. To do that, he says, a much bigger, controlled study is necessary. Barring that, he says, the current study provided insufficient detail about brands, models, and settings of the washers and dryers used even to allow independent researchers to reproduce it.

“You don’t really see trends that are easily explained” in the data, Smith says. For instance, certain chemicals appeared at higher concentrations in the vent air when detergent alone was used than when both it and dryer sheets were, or when no products were used than when one or both were used. He also points out that only a handful of the identified chemicals—including acetaldehyde, acetone, and ethanol—are fragrance ingredients, and that some of these occur naturally.

Steinemann agrees that more work is needed. Her next step, she says, will be a study comparing dryer-vent emissions during the use of fragranced and fragrance-free products.

The International Fragrance Association has published a list of more than 3,100 materials reported by manufacturers to be used in fragrance compounds.^16^ However, neither the Food and Drug Administration^17^ nor the Consumer Product Safety Commission^18^ require that individual fragrance ingredients be listed on labels or Material Safety Data Sheets. Steinemann and colleagues note that, of the VOCs they identified, only ethanol was listed on the label or sheet of either product.^1^
